# Effects of phantom microstructure on their optical properties

**DOI:** 10.1117/1.JBO.29.9.093502

**Published:** 2024-05-06

**Authors:** Jošt Stergar, Rok Hren, Matija Milanič

**Affiliations:** a “Jožef Stefan” Institute, Ljubljana, Slovenia; b University of Ljubljana, Faculty of Mathematics and Physics, Ljubljana, Slovenia; c Institute of Mathematics, Physics, and Mechanics, Ljubljana, Slovenia; d Syreon Research Institute, Budapest, Hungary

**Keywords:** hyperspectral imaging, spectroscopy, microscopy, tissue phantoms

## Abstract

**Significance:**

Developing stable, robust, and affordable tissue-mimicking phantoms is a prerequisite for any new clinical application within biomedical optics. To this end, a thorough understanding of the phantom structure and optical properties is paramount.

**Aim:**

We characterized the structural and optical properties of PlatSil SiliGlass phantoms using experimental and numerical approaches to examine the effects of phantom microstructure on their overall optical properties.

**Approach:**

We employed scanning electron microscope (SEM), hyperspectral imaging (HSI), and spectroscopy in combination with Mie theory modeling and inverse Monte Carlo to investigate the relationship between phantom constituent and overall phantom optical properties.

**Results:**

SEM revealed that microspheres had a broad range of sizes with average (13.47±5.98)  μm and were also aggregated, which may affect overall optical properties and warrants careful preparation to minimize these effects. Spectroscopy was used to measure pigment and SiliGlass absorption coefficient in the VIS-NIR range. Size distribution was used to calculate scattering coefficients and observe the impact of phantom microstructure on scattering properties. The results were surmised in an inverse problem solution that enabled absolute determination of component volume fractions that agree with values obtained during preparation and explained experimentally observed spectral features. HSI microscopy revealed pronounced single-scattering effects that agree with single-scattering events.

**Conclusions:**

We show that knowledge of phantom microstructure enables absolute measurements of phantom constitution without prior calibration. Further, we show a connection across different length scales where knowledge of precise phantom component constitution can help understand macroscopically observable optical properties.

## 1 Introduction

Tissue-mimicking phantoms are essential to the calibration, characterization, verification, and quality control of devices in medical physics. Their primary purpose is to simulate the physical properties of living tissue and facilitate the goals mentioned earlier. The secondary use of phantoms is in validating and studying algorithms that simulate biological systems and are used to evaluate results. Their use is already well-defined and regulated in fields, such as medical imaging and radiotherapy,^1^ while biomedical optics phantoms are an active research area. The development of reliable phantoms is, in fact, a key stepping stone in the development of quantitative imaging biomarkers^2^ and in the standardization of optical imaging methods,^3^^,^^4^ which are needed for the broad adoption of novel optical methods. Many optical tissue phantoms with different recipes have been designed and proposed throughout recent years, with an ongoing effort to set the design standards and systematically approach the characterization, verification, and validation of these phantoms.^5^ Here, we present a short overview of some of the different tissue phantoms successfully used in biomedical optics in recent years. The first group consists of liquid phantoms,^6^^,^^7^ which are usually based around a liquid scattering base, such as Intralipid or milk,^8^ with the addition of a pigment, such as India ink. They are affordable and straightforward to prepare but are challenging to use in complex geometries while also exhibiting a short shelf-life and variations between different batches of input components. These characteristics usually limit their use because they can give rise to an erroneous evaluation of the scattering coefficient.^8^

Some shortcomings of the liquid phantoms are alleviated by phantoms based on the hydrogel. In addition to mimicking tissue optical properties, these phantoms can simulate the structure and other tissue properties.^9^^,^^10^ While presenting a more reliable alternative to liquid phantoms, their shelf life and stability are their main shortcoming.

Solid tissue phantoms were developed to alleviate short shelf life while simultaneously offering the possibility of creating complex, multi-layered phantoms. Typically, they are based around a wax or a polymer matrix, such as PDMS, with additional components that assure appropriate scattering and absorption.^11^^–^^16^ An exciting development in solid tissue phantoms is the advent of three-dimensional printing technology, which allows the production of complex geometrical features found in realistic tissue samples.^17^^–^^19^ These phantoms significantly improve the shelf-life and stability of manufactured phantoms, but they often include monodisperse microspheres as a scattering component, thus increasing the price dramatically. Furthermore, clear polymers are often not sufficiently transparent in the biomedical optical range of visible and near-infrared light.^20^

A recent contribution to the variety of stable, solid tissue phantoms was the introduction of PlatSil SiliGlass silicone rubber phantoms,^20^ in which black silicone pigment serves as the absorber and silica microspheres as the scatterer. The advantages of the SiliGlass silicone rubber over other silicone-based rubbers are well documented. They include better control over curing and, thereby, better control of absorption and scattering properties, lower intrinsic absorption, and longer shelf life compared to other solid phantom substrates.^12^^,^^18^ The optical characterization performed by Konugolu Venkata Seker et al.^20^ demonstrated the reproducibility and homogeneity of SiliGlass phantoms while allowing for the selection of absorption and reduced scattering coefficients.

Naglič et al.^21^ complemented the study of Konugolu Venkata Seker et al.^20^ by improving the technological preparation of the phantoms and showing that (i) their refractive index was independent of the content of hollow silica spheres and black pigment and that (ii) the attenuation coefficient exhibited a linear relationship with the concentration of the black pigment. However, they found that the scattering component of the black pigment particles also contributed to the total attenuation coefficient and concluded that while PlatSil SiliGlass phantoms showed much promise, their optical properties would still need to be further characterized by advanced methodologies.

The primary objective of this study was to assess the optical and microscopic properties of PlatSil SiliGlass phantoms and their relationships. Although silica microspheres are non-monodisperse, their size distribution is closer to realistic tissue with a pronounced size ultrastructure.^22^ Furthermore, although challenging to study, the polydisperse nature of the scattering component makes it affordable and enables the creation of larger phantoms approximating semi-infinite mediums. To investigate the phantom optical properties, we examined multiple significant components of the phantom using different modalities and methods. First, the structure of the scattering component was studied using scanning electron microscopy (SEM) to determine the actual size distribution and microsphere structure. Absorption properties of the pigment and SiliGlass polymer were then measured using a laboratory spectrometer. Based on this data, a theoretical model was built using Mie theory^23^ and utilized to calculate the scattering coefficient of the phantom. Furthermore, all the results were applied to measured reflectance spectra of the phantom, enabling the determination of constituent volume fractions employing inverse Monte Carlo. Finally, the effects of single scattering and pigment aggregation were observed using hyperspectral microscopy.

## 2 Methods

### 2.1 Tissue Phantoms

The preparation of the SiliGlass phantoms was based on a report by Konugolu Venkata Seker et al.^20^ The exact recipe for preparation, including rigorous sonification, degassing, and temperature-controlled curing of the phantom, is described in detail in a previous paper.^21^ The medium for the phantom was PlatSil SiliGlass (Polytek), and the dedicated black absorber was Polycraft Black Silicone Pigment (MB Fibreglass, United Kingdom). The scatterers were silica microspheres (No. 440345, Sigma-Aldrich) with an expected mean sphere diameter of 9 to 13  μm, as specified by the manufacturer. We used two low-absorption phantoms (Aa and Da) and two high-absorption phantoms (Af and Df). Approximate values of reduced scattering and absorption coefficient values obtained from the original paper^20^ are given in Table 1.

**Table 1:** Predicted approximate optical properties of the four manufactured SiliGlass tissue phantoms at 600 nm as given by the recipe.^20^

	$\mu_{a}(600\;\mathrm{nm})$ (${\mathrm{cm}}^{-1}$)	$\mu_{s}^{\prime }(600\;\mathrm{nm})$ (${\mathrm{cm}}^{-1}$)
Aa	≈0.1	≈5
Af	≈0.9	≈5
Da	≈0.1	≈22
Df	≈0.9	≈22

During the phantom manufacturing process, all the components were weighted, along with the residual material in the mixing containers and on mixing utensils, to obtain exact mass fractions of individual components. Here, we assume that the mass does not change during curing. In addition, the density of the spheres was measured by weighting and mixing with water of pure microsphere scatterer. The determined ratio of densities was $\rho_{\text{sphere}}=1.196(1\pm 0.025)\rho_{\text{SiliGlass}}$. This ratio was used to convert between volume and mass fractions while accounting for the overall phantom density change due to the inclusion of scatterers. The dimensions of the produced phantoms before further processing conformed to the sizes of the urine cups (A5-50.20.18APL, Mikro + Polo, Slovenia) used for the production. The phantoms were thus cylindrical, with a diameter of 50 mm and heights of about 35 mm.

After manufacturing the phantoms, they were cut in half and wet-sanded for macroscopic imaging. This cut-and-sanding process prevented both imaging of edges of the phantom near the container, where the concentrations of constituent components could be off, and removed most of the surface texture artifacts due to cutting. The manual wet sanding was performed with a silicon carbide wet sanding paper (CP918A, VSM, Germany) with progressive grits P320, P500, and P1200. The sanding patterns were randomized to minimize sanding traces. After sanding, the phantoms were washed thoroughly and inspected for residue under a microscope. Half of the phantom was cut into arbitrary long square rods with a cross-section of ∼1  cm by 1 cm and stored for further microscopy imaging. The sanded phantoms were placed on the imaging table of the system with the sanded side facing the camera and imaged in the reflectance geometry.

**Fig. 1:**
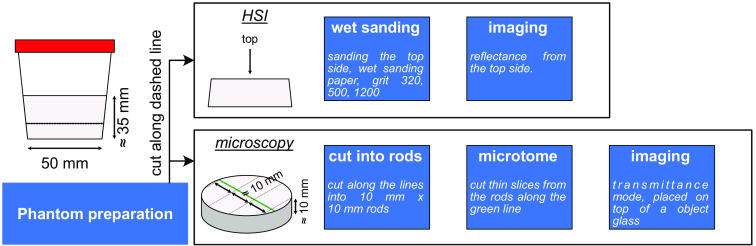
Workflow of phantom post-production. Phantoms were first cut along a line 1 cm from the bottom. The top part was used for imaging using a laboratory HSI system, while the bottom part was used for microscopy. The phantom for HSI was sanded with wet sanding paper on the cut side and imaged in reflectance mode. The phantoms for microscopy were first cut into square rods and then sliced into thin slices using a handheld microtome. Imaging was performed on a microscopy object glass in transmission geometry.

For the microscopic imaging, thin slices of the phantoms were cut from the rods obtained as leftovers after phantom cutting for macroscopic imaging. The samples were slit using a fresh cardboard knife blade using a handheld microtome; during this process, the samples were supported in the microtome utilizing a piece of hard plastic to prevent bending in the direction of cutting and consequent crumbling. The slices were then placed on a clean microscope object glass and imaged in the transmittance geometry. The sample thickness was measured by focusing the microscope on the glass slide and setting the z-position reader to zero. The area that was to be imaged was focused, and the offset of the z-axis gave its precise thickness: (770±10)  μm for phantom Aa, (580±10)  μm for phantom Af, (250±50)  μm for phantom Da, and (240±60)  μm for phantom Df. Since the cut was imperfect, the difference in thickness between different sides of the sample was given as the uncertainty. The Da and Df samples were cut thinner than the Aa and Af samples, resulting in more significant thickness variations. The whole process of phantom preparation for imaging is schematically presented in Fig. 1.

### 2.2 Hyperspectral Imaging and Data Normalization

Hyperspectral imaging (HSI) was performed on two size scales to examine the effects of the phantom microstructure on its optical properties, described in detail below.

Average reflectance spectra of tissue phantoms were obtained using our custom-developed macroscopic laboratory HSI system.^24^ In brief, the system is based around an imaging spectrograph (ImSpector V10e, Specim, Finland) and a custom broadband LED light source enabling operation in the wavelength range between 400 and 1000 nm with a spectral resolution of 2.9 nm. Multiple different objectives can be used with the system, which results in the spatial resolution of the system at 500 nm of 0.3 and 0.1 mm for 17 and 50 mm lenses, respectively. The system employs a push-broom methodology, whereas the reflectance signal is acquired along a line scanned perpendicularly over the sample. The measured intensity is divided by the reflectance recorded on a white reference standard (WS1, Labsphere Inc.) with a 99.9% reflectance, obtaining normalized reflectance $R_{x,y}(\lambda )$ in the form of

$$R_{x,y}(\lambda )=\frac{I_{x,y}^{\mathrm{meas}}(\lambda )}{I_{x,y}^{ref}(\lambda )},$$ (1)

where $I_{x,y}^{\mathrm{meas}}(\lambda )$ is the measured intensity reflected from the phantom and $I_{x,y}^{\mathrm{ref}}(\lambda )$ is the measurement performed on a white reference. Note that the dark current is not included in this equation because the camera already compensates for it.

The phantoms were also examined on a micro-scale using thin phantom slices where only a handful of scattering events occurred. To this end, our custom hyperspectral microscope was used.^25^ The microscope operates in the filtered regime, acquiring a sample transmittance at one wavelength at a time. Scanning the whole wavelength range between 450 and 750 nm enables the acquisition of an entire hyperspectral cube with a spectral resolution of 2.5 nm and a maximal attainable spatial resolution of 1.3  μm with a 50× magnification objective. The system uses a custom-developed monochromatic light source based on a Czerny–Turner configuration monochromator with built-in on-line spectroscopic monitoring. All images were recorded with a spectral step of 1 nm and a camera integration time of 1 s; the images were binned in each spatial direction by a factor of 2. After the binning, the detector’s dark current was subtracted from the sample and reference measurements. The sample measurements were divided by the unobstructed beam reference image acquired using the same system settings, resulting in the normalized transmittance hyperspectral microscope images:

$$T_{x,y}(\lambda )=\frac{I_{x,y}^{\mathrm{meas}}(\lambda )-I_{x,y}^{\text{dark}}}{I_{x,y}^{\mathrm{ref}}(\lambda )-I_{x,y}^{\text{dark}}},$$ (2)

where $T_{x,y}(\lambda )$ is the transmittance, $I_{x,y}^{\mathrm{meas}}(\lambda )$ is the measured intensity, $I_{x,y}^{\text{dark}}$ is the dark current, and $I_{x,y}^{\mathrm{ref}}(\lambda )$ is the unobstructed beam white reference.

### 2.3 Microsphere Size Distribution Determination

Microscopic properties of the scattering elements in the phantoms govern the overall scattering properties. The main ingredients of the phantom that contribute to the scattering are the glass microspheres. While the pigment particles also scatter light, their concentration is typically much smaller than that of the glass spheres. In addition, air bubbles that remained trapped in the polymer could potentially contribute to scattering, but their presence was minimized as best as possible through rigorous vacuuming of the samples.

Microscopic properties of the spheres were assessed by a scanning electron microscope (SEM)^26^ Helios NanoLab 650 (FEI Company). A small amount (tip of a spatula) of glass sphere powder was placed on an adhesive imaging substrate and imaged at different magnifications; raw SEM images were preprocessed by locally enhancing the contrast using a MATLAB function *adapthisteq*. Afterward, circular objects within the image were detected using a MATLAB function *imfindcircles* with phase detection method, and their diameters were extracted. The spheres’ diameter was converted from pixels to μm using the scale bar included in the images.

### 2.4 Absorption Properties Measurement and Determination

Absorption properties of both the polymer material and absorption component were measured in the transmittance mode by placing the material in a standard transparent polystyrene cuvette (Makro PS cuvette, 2711110, Ratiolab, GmbH) on a laboratory spectrometer (Lambda 960, Perkin-Elmer). The absorption coefficient was calculated from the transmittance values following the procedure described by Li et al.^27^ The approach accounts for reflections from the cuvette walls by accounting for the medium and cuvette refractive index and absorption coefficient of the cuvette material. The refractive indices for polystyrene and SiliGlass were obtained from the literature.^21^^,^^28^^,^^29^ The absorption coefficient of the polystyrene was measured by using the transmittance model on an empty cuvette with the expected content transmittance of 1.

### 2.5 Calculation of Scattering Coefficient

The scattering coefficient and anisotropy were calculated for full and hollow quartz glass spheres using Mie theory.^30^ A C programming language routine by Prahl^31^ was used for full spheres, and a routine from Bohren and Huffman in Fortran programming language *BHcoat*^30^ was used for hollow glass spheres. Hollow glass spheres were simulated as a quartz spherical shell surrounding an air bubble. For all the simulations, the refractive index of the surrounding material was set to that of SiliGlass, as obtained from the literature.^21^ The refractive index of air was taken to be n=1, and the refractive index of ${\mathrm{SiO}}_{2}$ was obtained from the literature.^32^^,^^29^ The result of the simulation is the scattering efficiency $Q_{s}$ of a sphere, which is related to the scattering cross-section of a particle $C_{s}$ through its geometrical cross-section S as

$$C_{s}(\lambda )=S\;Q_{s}(\lambda )=\frac{\pi d^{2}}{4}Q_{s}(\lambda ),$$ (3)

where d is the diameter of the sphere.^23^ The scattering coefficient $\mu_{s}(\lambda )$ is then determined by multiplying the cross-section by the volume fraction of the scatterers:^22^

$$\mu_{s}(\lambda )=\overset{˜}{\rho }C_{s}(\lambda ),$$ (4)

where $\overset{˜}{\rho }$ is the volume fraction of the spheres. Given the relatively low volume fractions of the scattering components (about 10%), a non-interacting regime was assumed to enable the recalculation of the scattering coefficient by simple renormalization. To obtain the scattering coefficients of individual particles presented in this work, the scattering coefficient was calculated by setting the volume density to $\tilde{\rho }=0.1$, corresponding to a 10% volume fraction. When treating the mixture of different scatterers, the $\tilde{\rho }=1$ was used to calculate the scattering coefficient of individually sized pure scatterers and then weighted by the volume fractions of different sizes, resulting in the scattering coefficient of the mixture through summation for volume fraction of the mixture $\tilde{\rho }=1$ due to the normalization of volume fraction distribution to unity. Note that these steps are performed in the data processing after the calculation of scattering efficiency $Q_{s}$ by Mie theory and can be renormalized for different volume fractions at any time. In the case of higher scatterer volume fractions, an interaction term would have to be added to Eq. (4), which would decrease the values for densely packed particles.^22^ For lower volume fractions, the scattering coefficient can then be determined by multiplying the $\tilde{\rho }=1$ pure scattering coefficient with the percent value of the scatterer volume fraction.

In addition to the scattering coefficient $\mu_{s}$, the scattering anisotropy g was also calculated. The *BHcoat* routine used for the hollow spheres does not support the calculation of the scattering anisotropy, so it was approximated by simulating just the air bubble (the hollow center of the sphere) surrounded by the SiliGlass, assuming similarity of refractive indices between SiliGlass and silica.

### 2.6 Inverse Problem Solver

With known optical properties of pure phantom constituents, extracting volume fractions of individual components from the transmittance or reflectance values is possible by solving an inverse problem. To this end, a modified version of the Monte Carlo multi-layer (MCML) on CUDA^33^^,^^34^ was used. A set of initial values was assumed, and a nonlinear fitting routine *lsqnonlin* in MATLAB (Mathworks) was used to compute the volume fractions of individual components iteratively. The reflectance values were fitted at 166 wavelength points between 420 and 980 nm. To evaluate the goodness-of-fit of the final results, three metrics were calculated based on the measured reflectance spectrum $R^{\text{measured}}(\lambda_{i})$ and Monte Carlo reflectance spectrum $R^{\mathrm{MCML}}(\lambda_{i})$. First, root-mean-square-error

$$\mathrm{RMSE}=\sqrt{\frac{1}{N}\overset{N}{\underset{i}{\sum }}[R^{\text{measured}}(\lambda_{i})-R^{\mathrm{MCML}}(\lambda_{i}){]}^{2}}$$ (5)

was calculated, as this is the standard metric of goodness-of-fit. Next, the wavelength interval was divided into two intervals, and for each one, the mean absolute deviation $\left|\overset{¯}{\Delta R}\right|$ and maximal absolute deviation max|ΔR| in units of reflectance were evaluated. Here, $\Delta R=R^{\text{measured}}(\lambda_{i})-R^{\mathrm{MCML}}(\lambda_{i})$ is the difference between the measured and MCML calculated reflectances.

### 2.7 Software Environment

The simulations, including routines for calculating the scattering coefficient and inverse Monte Carlo, were performed on a desktop computer with an Intel i5 CPU, 16 GB of RAM, and Nvidia GTX1050-Ti graphics cards under the Arch Linux operating system. The C and Fortran code was compiled using the GCC 10.2.0 compiler collection. CUDA implementation of Monte Carlo was compiled for CUDA 11.1, running under the driver version 455.45.01. The preparation of the simulation input files and parsing of the simulation outputs was conducted using Python 3.9.1, which was running a web server implemented in Flask that enabled the invocation of individual computation routines over HTTP requests. The data processing was performed on a laptop computer (Intel i5, 16 GB of RAM) using MATLAB R2020a (Mathworks) with Optimization Toolbox and Image Processing Toolbox.

## 3 Results

### 3.1 Absorption Properties

**Fig. 2:**
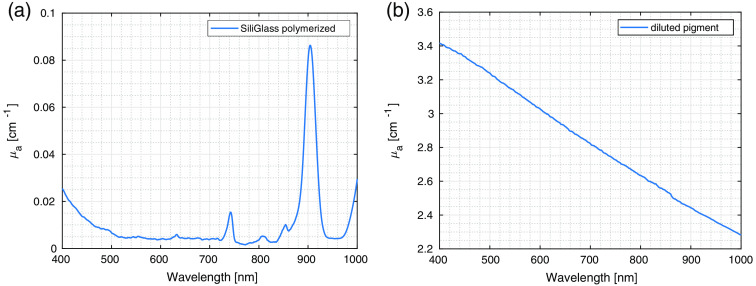
Measured absorption coefficients of (a) SiliGlass polymer and (b) diluted pigment.

The absorption properties were measured for the 1:2272 diluted pigment used to prepare the phantoms (termed the absorbing component or pigment in the continuation) and polymerized SiliGlass phantom medium. The absorption coefficients for both the pure polymerized polymer and pigment calculated from transmittances measured on the laboratory spectrometer are shown in Fig. 2. The values for absorption were compared to values given by Konugolu Venkata Seker et al.^20^ Series a phantoms have a reported $\mu_{a}(600\;\mathrm{nm})\approx 0.1\;{\mathrm{cm}}^{-1}$. For our measurements, mass fractions of diluted pigment were about (3.7±0.2)% resulting in $\mu_{a}=(0.11\pm 0.01)\;{\mathrm{cm}}^{-1}$, assuming the densities of the whole phantom are close to the absorber component. For phantoms f, the literature reports $\mu_{a}(600\;\mathrm{nm})\approx 0.9\;{\mathrm{cm}}^{-1}$. In our measurement, mass fractions of the diluted pigment were (33±1)% resulting in $\mu_{a}=(0.99\pm 0.03)\;{\mathrm{cm}}^{-1}$. From this comparison, the measured absorption coefficient agrees well with the previously reported approximate numbers within the uncertainties.

### 3.2 Microsphere Size Distribution

**Fig. 3:**
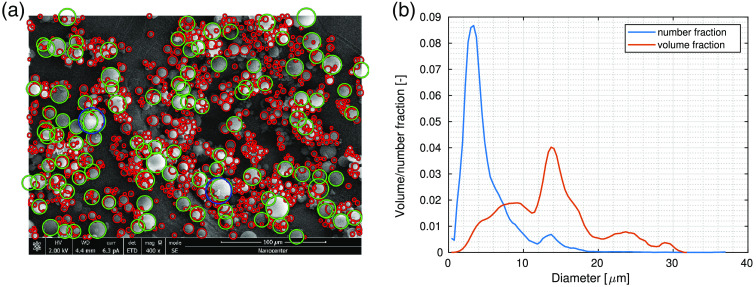
(a) An example of microsphere diameter detection from SEM images (400× magnification); (b) microsphere diameter distribution normalized both in terms of number and volume fraction.

The glass microspheres were imaged under SEM at four different magnifications, 80× to 3000×; in total, 12 images of different regions were acquired for microsphere size analysis. As expected from the manufacturers’ specifications, the images reveal a distribution of spheres of various sizes with some larger aggregates. Diameters of 8156 microspheres were determined from SEM images; an example of the process of diameter determination is shown in Fig. 3(a). To account for a large diameter variability among microspheres, images at different magnifications were used. Some spheres remained undetected, but visual inspection revealed that the detection probability was not related to the sphere’s size but to the sphere’s apparent brightness in the image. Based on this observation, undetected spheres should not introduce bias into the size distributions.

The microsphere size histograms were normalized to unity to obtain number fraction distribution. The volume fraction distribution was calculated by multiplying the number fractions by appropriate sphere sizes. The distribution was then normalized to unity and smoothed using a moving average filter in MATLAB, removing statistical noise due to the relatively small sample size. The resulting number and volume distributions are shown in Fig. 3(b), exhibiting two distinct peaks. Most spheres were relatively small, while a smaller number of larger spheres contributed prominently to the volume fraction. The average diameter of microspheres was (13.47±5.98)  μm when considering volume fraction distribution, which was close to the range of 9 to 13  μm given by the manufacturer.

**Fig. 4:**
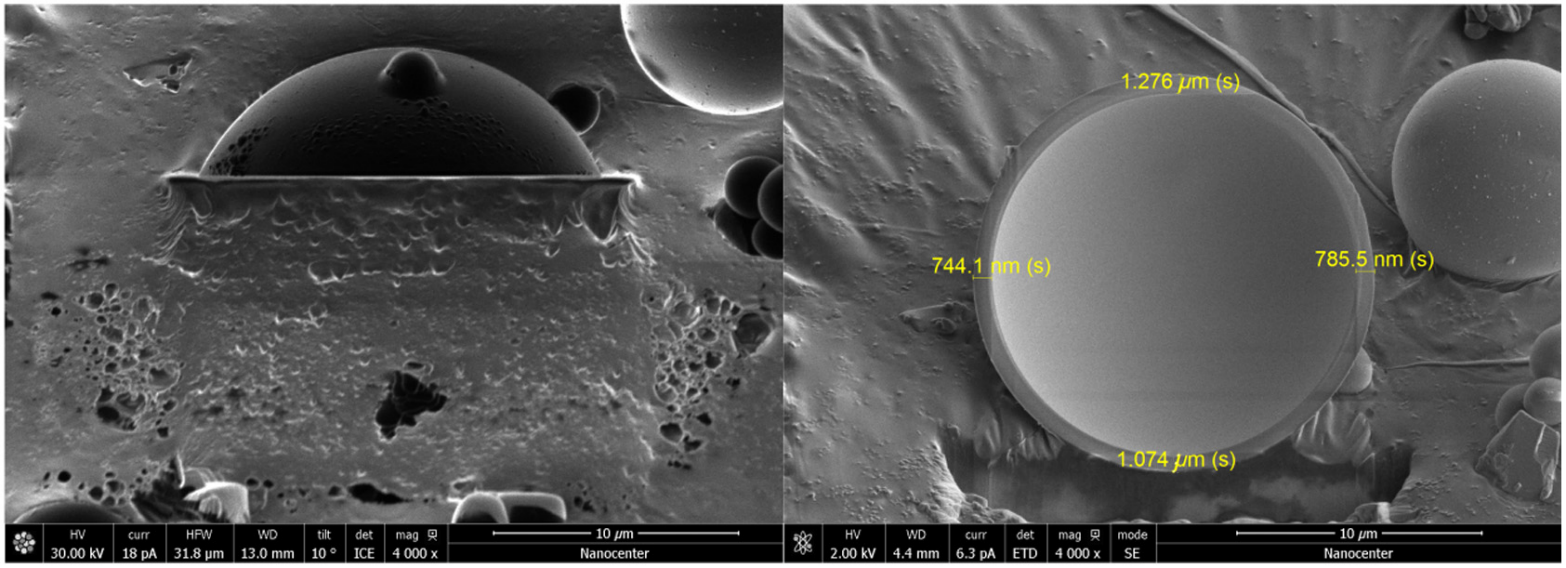
SEM image of a sphere cut in half by an FIB. A microsphere with a diameter of 18  μm, cut in half, is shown in the top and side view; magnification is 4000×.

The spheres could be hollow, so the wall thickness was introduced as another vital parameter besides the diameter. The wall thickness was measured by cutting four spheres of different diameters (3.4, 8.4, 18, and 24.6  μm) in half using a focused ion beam (FIB)^35^ integrated into the SEM device. After cutting the spheres, the resulting cross-section was imaged using SEM, with an example shown in Fig. 4. The smaller sphere (3.4  μm) was observed to be solid, while the largest sphere (24.6  μm) had a small air bubble that was not centered. For the medium-sized spheres (diameters 8.4 and 18  μm), an average wall thickness (0.9±0.2)  μm was observed. The number of spheres cut in half was limited due to the long time needed to cut the sphere in half and the limited time on the device.

### 3.3 Predicted Scattering Coefficient and Anisotropy

**Fig. 5:**
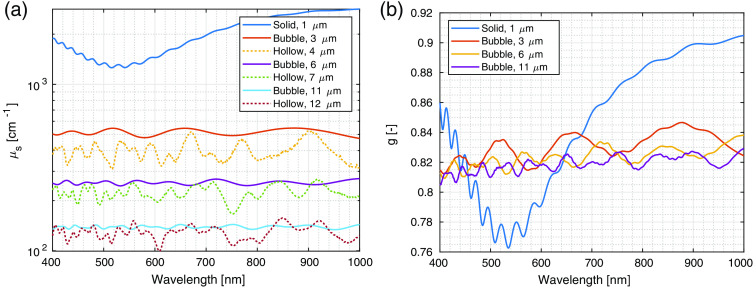
Scattering coefficient and anisotropy for some example spheres calculated using Mie theory: (a) scattering anisotropy of small solid glass spheres, hollow spheres consisting of an air bubble with a quartz glass shell, and air bubbles (e.g., hollow spheres without the glass shell) submersed in the SiliGlass polymer; (b) scattering anisotropy calculated for full glass spheres and air bubbles in a SiliGlass polymer. The size value in the legend is the radius of the simulated sphere. In the cases of hollow spheres, the simulated shell thickness is 1  μm so that the air volume is the same as in the case of the air bubble without the shell.

Based on the determined scatterer microstructure, Mie theory numerical calculations were performed. First, some representative examples from the distribution were selected and are shown in Fig. 5. In general, smaller spheres exhibited more pronounced changes in scattering coefficient across the observed spectral range, as expected from Mie theory. The difference between the hollow and full spheres was particularly interesting for larger spheres. Both full and hollow spheres exhibited similar scattering coefficient values with more significant deviations from the average value for hollow spheres.

A model that approximates the realistic properties of the scattering component was developed based on the measurements of sphere wall thickness. First, the wall thickness distribution was approximated to be box-shaped between 0.7 and 1.1  μm with a step size of 0.1  μm. For each wall thickness, scattering coefficients and anisotropies were calculated following the size distribution of spheres. For the calculations, the distribution was interpolated to 0.1  μm step size and renormalized to unity. As a verification, a denser distribution was also examined but showed no significant changes in the final scattering coefficient. The spheres with a radius smaller than the wall thickness were simulated as full glass spheres (denoted in the equations by omitting the wall thickness parameter $d_{\text{wall}}$). Due to the limitations of the numerical codes, scattering anisotropy was calculated for full glass spheres in the case of small spheres and as air bubbles with radius decreased by the wall thickness in the case of larger spheres. The whole process is summed up by the model equations:

$$\mu_{s}(\lambda )=\overset{1.1\;\mu m}{\underset{d_{\text{wall}}=0.7\;\mu m}{\sum }}0.2\underset{r}{\sum }\phi_{r}\{\begin{array}{ll} \mu_{s,r,d_{\text{wall}}}(\lambda ), & r>d\\ \mu_{s,r}(\lambda ), & r\leq d \end{array},$$

$$g(\lambda )=\overset{1.1\;\mu m}{\underset{d_{\text{wall}}=0.7\;\mu m}{\sum }}0.2\underset{r}{\sum }\phi_{r}\{\begin{array}{ll} g_{\text{bubble},r-d_{\text{wall}}}(\lambda ), & r>d\\ g_{\text{glass},r}(\lambda ), & r\leq d \end{array}.$$

**Fig. 6:**
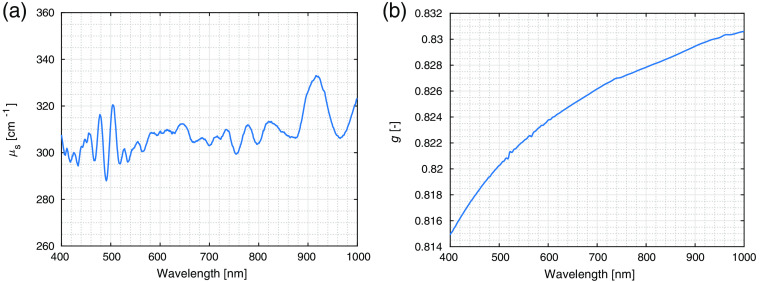
(a) Scattering coefficient and (b) scattering anisotropy calculated for the determined size distribution of spheres in a non-interfering regime, accounting for the distributed wall thickness. The values are calculated for a 10% volume fraction of the spheres.

An approximation of both scattering coefficient and scattering anisotropy for the size-distributed silica microsphere scattering component was calculated using the model equations and is shown in Fig. 6 for 10% volume fraction of the spheres.

The scattering coefficient values can be again related to the values reported in the literature.^20^ Given the 10% volume fraction of the scattering component in the simulations, the scattering coefficient for 1% sphere volume fraction would be $\mu_{s}(600\;\mathrm{nm},1\%)=31\;{\mathrm{cm}}^{-1}$ with the scattering anisotropy of g=0.824. The mass fractions of the scattering component determined during the preparation were (1.7±0.1)% and (7.5±0.1)% for phantoms in series Ax and Dx, respectively. Correcting for the densities of the spheres, we arrive at volume fractions of (1.5±0.1)% and (7.3±0.1)% for both phantoms. Estimating the reduced scattering coefficient as $\mu_{s}^{\prime }=(1-g)\mu_{s}$ gives values $\mu_{s}^{\prime }(600\;\mathrm{nm})=8.2(1\pm 0.1)\;{\mathrm{cm}}^{-1}$ and $\mu_{s}^{\prime }(600\;\mathrm{nm})=39.8(1\pm 0.1)\;{\mathrm{cm}}^{-1}$ for phantoms in A and D series, respectively. These results agree with the ones reported in the literature to the order of magnitude but exhibit deviations that could be due to the specifics of the hollow particle scattering phase function. For example, changing the scattering anisotropy to g=0.9 would cause the reduced scattering values to agree with those from the literature given in Table 1 perfectly.

Furthermore, seemingly exaggerated spectral features were observable between 400 and 500 nm. These could be attributed to a rather similar glass shell thickness in the case of hollow spheres. In general, if the shell thickness variations are larger than accounted for in this study, this would represent about 7% relative uncertainty in the presented data.

### 3.4 Determination of Component Volume Fractions

**Fig. 7:**
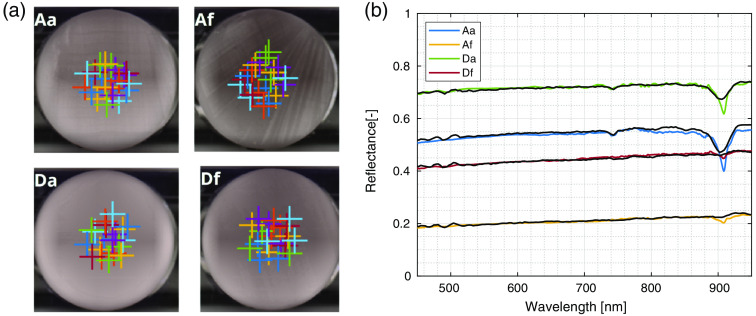
Measured and fitted spectra of tissue phantoms using a laboratory HSI system. (a) RGB projections of phantoms with sampling points used to calculate average reflectance spectra are denoted. Please note that the apparent inhomogeneity in the images results from RGB projection and aggressive histogram equalization in the visualization software. (b) The calculated average reflectance spectra for all four phantoms are displayed (colored lines) along with the results of inverse Monte Carlo fitting (black lines). Standard deviations of spectra for the sampled points are smaller than the line thickness.

The knowledge of sphere microstructure enabled numerical calculation of the predicted scattering coefficient. The measured absorption properties of the phantom constituents made it possible to determine the component volume fractions based on the HSI-measured spectra. The four phantoms were measured using the HSI system. Spectra were averaged from 20 points in the center of the phantom, as shown in Fig. 7(a). Average reflectance spectra, normalized to a white reference, are shown in Fig. 7(b) as colored lines with noticeable oscillations around 500 nm and between 800 and 900 nm. A comparison of reflectance spectra with the numerically calculated scattering coefficient in Fig. 6 confirms that similar shapes are observed.

Beyond qualitative comparison, the known sample microstructure made it possible to determine absolute volume fractions of phantom components. This was achieved by fitting the measured average reflectance spectra using the MCML on CUDA algorithm. The measured absorption and calculated scattering spectra were used as inputs, while the volume fractions of constituent components were left as free parameters. Graphically, the results of this inverse problem are shown in Fig. 7(b) with full black lines, achieving a good agreement between the measured and fitted spectra. Note that the oscillations around 500 nm are slightly exaggerated in the fit results, which is attributed to the limited knowledge of sphere wall thickness. Numerically, the results also exhibit good agreement between the experimental and theoretical values, as indicated by the deviations of the fit from the measured values and low RMSE numbers in Table 2. Numerical results for volume fractions of scattering and absorption components obtained by this process are presented in Table 3 and again show good agreement within the uncertainties of the method.

**Table 2:** Evaluation of goodness-of-fit for Monte Carlo fitting. For each phantom, the absolute mean deviation of the reflectance, as well as the maximal absolute value deviation, is given for two wavelength windows. In the last column, the root-mean-square-error of the fit for the whole range between 420 and 980 nm is reported.

	420 to 850 nm		850 to 980 nm		RMSE (−)
	$\left|\overset{¯}{\Delta R}\right|$ (%)	max|ΔR| (%)	$\left|\overset{¯}{\Delta R}\right|$ (%)	max|ΔR|	
Aa	0.5	1.6	1.0	7.9	0.0099
Af	0.2	0.9	0.3	1.2	0.0032
Da	0.3	1.1	0.7	5.1	0.0063
Df	0.4	1.2	0.8	1.7	0.0061

**Table 3:** Values of mass fractions obtained by weighting during the preparation for absorber $w_{\mathrm{abs}}$ and scatterer $w_{\mathrm{scat}}$ in comparison to the mass fractions of the absorber ${\tilde{w}}_{\mathrm{abs}}$ and scatterer ${\tilde{w}}_{\mathrm{scat}}$, as obtained by solving the inverse problem using computed scattering and measured absorption coefficients with the Monte Carlo method.

	$w_{\mathrm{abs}}$ (%)	${\overset{˜}{w}}_{\mathrm{abs}}\;$ (%)	$w_{\mathrm{scat}}$ (%)	${\overset{˜}{w}}_{\mathrm{scat}}$ (%)
Aa	3.8±0.4	5.0±0.7	1.7±0.2	2.1±0.3
Af	34±3	44±6	1.7±0.2	2.0±0.3
Da	3.5±0.4	3.7±0.5	7.5±0.8	6.7±0.9
Df	32±3	32±4	7.6±0.8	7.0±0.9.

### 3.5 HMI Evaluation of Tissue Phantoms

**Fig. 8:**
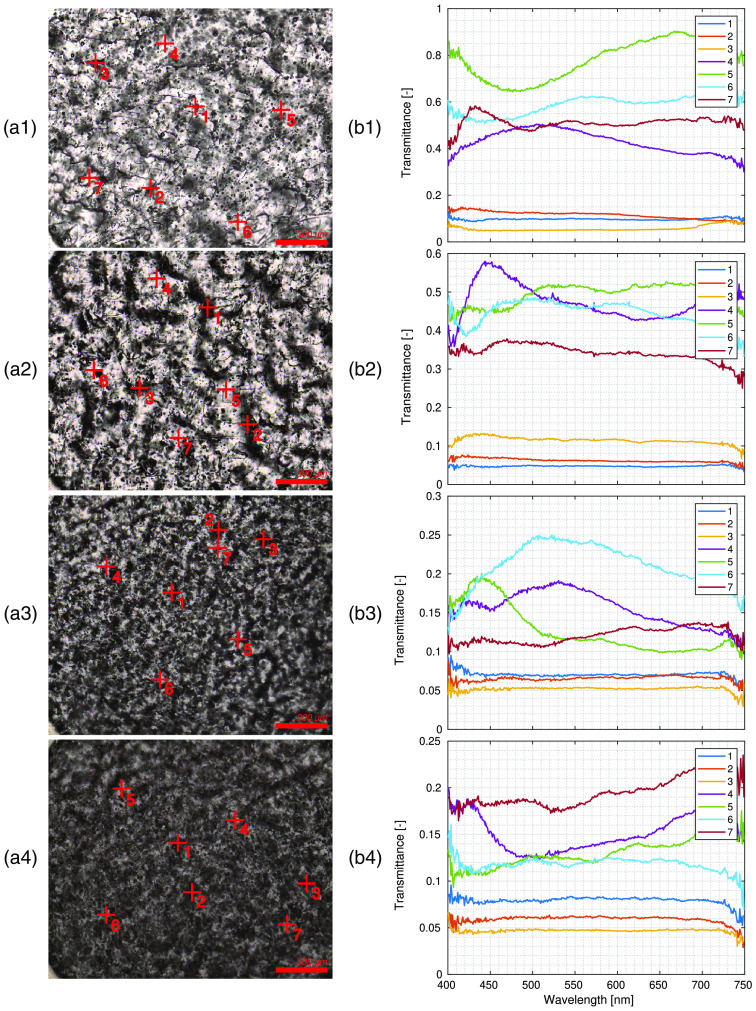
Microscopic HSI of phantoms. In column (aX), RGB projections of the hyperspectral image (10x magnification) are shown. In column (bX), the spectra sampled at the sampled points denoted in column (aX) are shown. The first row (a1), (b1) shows phantom Aa, the second row (a2), (b2) phantom Af, the third row (a3), (b3) phantom Da, and the fourth row (a4), (b4) phantom Df. Locations 1 to 3 on each image correspond to the dark areas with absorber agglomerates, while areas 4 to 7 are sampled on the brighter spots near the scattering spheres. Spectral features due to the Mie scattering are observable in feature-rich spectra in those points. All spectral plots share the same wavelength axis at the graphs’ bottom. All spectra are binned over 16 pixels of the original image centered around the point marked in (aX).

First, RGB projections of the normalized images were calculated by projecting the transmittance spectra to CIE XYZ color space and applying a standard D65 illuminant; the resulting images are shown in Fig. 8(a). The larger spheres visible in the images were dispersed equally over the sample, but aggregates of spheres, which were already observed in SEM images, are also visible using the HMI as irregularly shaped small inclusions. It appears that despite the rigorous sonification and mixing during the phantom preparation, not all the clusters of microspheres were successfully broken.

The transmittance spectra were sampled from the hyperspectral images at multiple sites to analyze different phantom parts [Fig. 8(b)]. The RGB images and corresponding spectra revealed two distinct area types of the phantom. The locations of darker and brighter areas are denoted in Fig. 8 with 1-3 and 4-7, respectively.

The darker areas, rich with the absorbing material, exhibited low transmittance, and it is evident that the ink particles were not dispersed equally over the whole phantom but were agglomerated. Only minor differences in transmittance values between high-absorption and low-absorption phantoms were observed in these darker areas; furthermore, transmittance spectra were practically constant in these areas. Both observations indicate that most of the light in these areas was absorbed over the whole wavelength range. It is important to note that under close examination, the darker areas corresponded to the rougher parts of the sample in some cases. This raises two important conclusions: (1) the presence of the pigment introduced a defect in the silicon matrix that causes the phantoms to break more frequently along areas rich in the absorber, and (2) the results presented for the darker areas could be, to varying degrees, also impacted by the uneven surface.

In brighter areas, the transmission spectra for each phantom exhibited a strong dependence on the wavelength, indicating the contribution of single scatterers to the spectrum. Since the thickness of the sample was small, the number of scattering events was likely low. Therefore, the characteristic Mie shape of scattering from a single particle instead of the average scattering was to be expected, which was confirmed by the oscillatory spectra.

## 4 Discussion

Stable and robust tissue-mimicking phantoms with easily and predictably adaptable optical properties are essential in biomedical optics. This paper presents an overview of the microscopic properties of scattering component in the proposed SiliGlass phantom. We explore the implications of the microscopic phantom structure on the optical properties of the phantoms. The macroscopically observed spectra are compared to theoretically predicted scattering properties of the microsphere by fitting experimental spectra with an inverse Monte Carlo algorithm. The effects of microscopic structure on the transmittance of thin phantom slices are further examined using hyperspectral microscopy. The results show that knowledge of phantom microstructure can be used to determine individual mass fractions of components that agree with the ones obtained by weighting during the phantom preparation.

The calculated values of the optical properties generally agree with the previous studies, except for reduced scattering. As pointed out earlier, reduced scattering is especially sensitive to the value of scattering anisotropy, which significantly depends on the wall thickness of the microspheres. This was, incidentally, also the parameter that was determined with the least accuracy due to the long time needed to process a single sphere. Although the scattering anisotropy is only estimated in the scope of this paper, it is essential to note that the benefit of theoretically calculated scattering coefficient is apparent because it enables quantitative measurement of the sphere mass fraction based on the reflectance spectra. In the future, the thickness of the glass walls could, and in fact should, be measured for a larger sample size, which will ultimately fully describe the optical properties of SiliGlass phantoms. In general, to achieve this objective, both the shell thickness and the fraction of the hollow spheres must be assessed. Given the complexity of slicing the spheres, only a limited number could be cut to obtain a better statistic on the wall thickness and then infer the volume fraction of the hollow spheres using sample density measurements. Another interesting direction is the development of a computational code that would also calculate the anisotropy for the hollow spheres, thus providing insights into disparities in reduced scattering coefficient between the previously reported approximate results and the results of this study.

A notable aspect of the presented results is the non-flat nature of the scattering coefficient. When comparing the calculated scattering coefficients for full and hollow spheres, an increase in the relative prominence of spectral features can be observed for the hollow spheres. We attribute these oscillations to the thin shell of glass that constitutes the hollow sphere. Because the thickness of the glass shell is similar between the spheres according to our measurements, it is anticipated that the contributions of the shells do not average out as much as oscillations due to the bulk Mie scattering. In contrast, within realistic biological tissue, the diversity of the ultrastructure is more pronounced, and thus no such prominent spectral features are expected. This, in effect, further underlines the importance of insight into the microscopic structure of samples when treating their scattering properties.

The accurate depiction of the microscopic structure of the phantoms carries important implications for the reproducibility and repeatability of phantoms based on the polydisperse scattering components, as demonstrated by this study. Given the absence of detailed specifications regarding the microscopic structure of such scattering spheres, discrepancies may arise between different batches of the spheres. This reinforces the importance of studying phantom component microstructure to ensure that phantoms are, in fact, performing as expected.

One of the challenges, already emphasized by Naglič et al.,^21^ is the prevention of agglomerates. Both SEM and HMI revealed the presence of agglomerated scattering and absorption components. In the development of predictable tissue phantom recipes, preventing such agglomerates presents a unique challenge that must be solved in the future. Alternatively, the findings of this study could be expanded in the future, establishing the inhomogeneity of the optical properties as an integral component of the phantom itself. Such characterization could still serve the purpose of standardization through the analysis of not only absolute optical properties but also their differences when testing novel imaging methods. This, in fact, brings such phantoms closer to mimicking realistic tissues with diverse structural properties. Interestingly, absorber agglomerates can be observed in the HMI transmittance spectra. While the brighter areas exhibit diverse transmittance values per single particle scattering, the dark regions exhibit similar, almost constant trends. Furthermore, the transmittance in these areas is seemingly independent of the absorber concentration, indicating that most of the light is totally absorbed in these regions, whereas the fully dissolved pigment contributes less to the observed decreased transmittance in the brighter areas of the image. To further explore this and mitigate the possible effects of surface coarseness due to cutting, the phantoms could be prepared as thin slices on a glass substrate. In this case, though, it is important to verify that the structure of such phantoms is, in fact, comparable to the bulk phantoms. During the study presented in this paper, we observed that the concentration of the scattering component is lower at the boundaries of the phantom than in the bulk, which was also the reason for undertaking the phantom cutting.

The results presented in this paper can first and foremost be interpreted as a study of phantom microstructure and its consequences on phantom optical properties. Nonetheless, the observed relationships could be expanded upon and clarified by increasing the number of phantoms studied in the future. Additional relationships between the phantom ingredients and resulting optical properties could be explored using different scattering components. Combined with macroscopic imaging, future studies could, in principle, help guide the development of reliable, stable, and repeatable tissue phantoms for the standardization of optical imaging methods in biomedicine. The diameter distribution of scattering phantom inclusions presented in this paper could be used in the future to develop and study coupling between the microstructure and resulting optical properties of phantoms and possibly tissues by methods such as the presented numerical Mie theory simulations.

Some of the problems of the phantom presented in this study, namely the difficulty in determining the precise scattering values, could be, in principle, solved by using monodisperse or very small scatterers. Although such solutions would alleviate the problems of size ultrastructure, they would increase the price in the case of monodisperse spheres or deviate from the regime of biological tissues with rich ultrastructure in the case of small Rayleigh regime scatterers. Using a scattering component with a complex ultrastructure that approaches tissue more closely seems prudent in this light. However, additional research is still needed to describe the wall thickness fully and thus completely characterize the phantoms.

## 5 Conclusions

In the scope of this study, we examined the microscopic structure of solid SiliGlass-based tissue phantoms and its effects on the observable macroscopic properties. We presented the measurement of the absorption coefficient of the dye and the constitution of the silica micro-sphere scattering component. We further studied the scattering properties of the spheres and observed the effects of the hollow sphere on the scattering coefficient using Mie theory-based numerical calculations. We have shown a good agreement with the existing absorption coefficient measurements in the literature while also observing the significant effect of sphere hollowness primarily on scattering anisotropy. We have performed the microscopic and macroscopic HSI of phantoms. The determined optical properties, based solely on the study of individual components, produced quantitative mass fractions of phantom components that agree with the measurements of component masses. Furthermore, the optical properties resulting from this study were already successfully applied in studies performed by our group to improve the treatment of hyperspectral image correction when imaging curved samples.^36^ In addition, we have shown the significance of sample microstructure that is further amplified by the few-scattering regime when using hyperspectral microscopy.

The significance of the presented study is thus twofold. First, it enables the extraction of quantitative sample properties without prior calibration. Second, it demonstrates a successful case of translating complex sample microscopic structures to optical properties. The results stress a critical aspect of optical imaging; despite the limited imaging resolution, the spectral information encodes a wealth of information about sample structure even below the resolution limit of the used system.

An interesting future direction is the complete characterization of sphere wall thickness and rigorous calculation of the scattering phase function that would completely characterize this and similar phantoms. We strongly believe that future research in this direction can improve the characterization of a phantom that might seem inappropriate but is closer to realistic tissue than simpler, monodisperse sphere phantoms and thus provide a powerful tool for both imaging system characterization as well as a playground for testing different numerical algorithms without oversimplification.

## Data Availability

The data supporting this article’s findings are available upon reasonable request from the corresponding author.
